# Supervised learning algorithm for analysis of communication signals in the weakly electric fish *Apteronotus leptorhynchus*

**DOI:** 10.1007/s00359-023-01664-4

**Published:** 2023-09-13

**Authors:** Dávid Lehotzky, Günther K. H. Zupanc

**Affiliations:** Laboratory of Neurobiology, Department of Biology, Northeastern University, 360 Huntington Ave, Boston, MA 02115 USA

**Keywords:** Signal analysis, Artificial intelligence, Supervised learning, Chirping behavior, Weakly electric fish, *Apteronotus leptorhynchus*

## Abstract

Signal analysis plays a preeminent role in neuroethological research. Traditionally, signal identification has been based on pre-defined signal (sub-)types, thus being subject to the investigator’s bias. To address this deficiency, we have developed a supervised learning algorithm for the detection of subtypes of chirps—frequency/amplitude modulations of the electric organ discharge that are generated predominantly during electric interactions of individuals of the weakly electric fish *Apteronotus leptorhynchus*. This machine learning paradigm can learn, from a ‘ground truth’ data set, a function that assigns proper outputs (here: time instances of chirps and associated chirp types) to inputs (here: time-series frequency and amplitude data). By employing this artificial intelligence approach, we have validated previous classifications of chirps into different types and shown that further differentiation into subtypes is possible. This demonstration of its superiority compared to traditional methods might serve as proof-of-principle of the suitability of the supervised machine learning paradigm for a broad range of signals to be analyzed in neuroethology.

## Introduction

Signals as vehicles for transmission of information from a sender to a receiver play a pivotal role in animal communication (Bradbury and Vehrencamp 2011). Broadcasting of signals is mediated by a variety of sensory channels, such as visual, acoustic, tactile, chemical, and electric. Diversity of signals, either within one sensory modality or by activation of several sensory channels, enables animals to use different signals for different behavioral functions. Within one sensory modality, signal diversity is often achieved by modulation of a generic type of signal. For example, different acoustic signals can be produced by temporal frequency and amplitude modulations, and even rather subtle differences can have profoundly different functional effects (Schwartz et al. 2007; Feng et al. 2009; Hechavarría et al. 2020).

While acoustic signals are displayed intermittently only (although sometimes for prolonged periods at high rates), some electric fishes produce a generic form of electric signal continuously throughout life. This group includes the brown ghost knifefish (*Apteronotus leptorhynchus*), a species of the taxonomic order Gymnotiformes that has been intensively studied as model organisms in ethology and neuroethology.

*Apteronotus leptorhynchus* generates such continuous electric discharges with its electric organ composed of modified axonal terminals of spinal motoneurons (for review see Zupanc and Bullock 2005). The synchronous depolarization of these so-called electrocytes produces electric pulses separated by short inter-pulse intervals. This results in the appearance of a continuous, wave-like signal, commonly referred to as electric organ discharge (EOD). The frequency at which the fish generates the EOD train is determined, in a one-to-one fashion, by the frequency of the neural oscillations of a central pattern generator in the medulla oblongata, the pacemaker nucleus. Within the species-specific frequency range of 650–1000 Hz, males discharge at higher frequencies than females, with little overlap between the sexes (Meyer et al. 1987; Zupanc et al. 2014). Owing to this sexual dimorphism, the EOD contains information about the sex of its sender.

Whereas the species as whole occupies a broad EOD frequency range, the frequency of the discharges of a given individual within this frequency band is highly constant, as indicated by the coefficient of variation [cv = (standard deviation / mean) $\times$ 100 (%)], which assumes values of less than 0.2% over 30-min (Eske et al. 2023). Nevertheless, transient modulations may occur, resulting in diversification of the generic EOD signal. The best-characterized type comprises chirps. In isolated individuals of *A. leptorhynchus*, chirps are very rarely produced, on average less than once per 10 min (Engler et al. 2000; Zupanc et al. 2001; Eske et al. 2023). However, during stimulation with the EODs of conspecific fish or with electric signals mimicking such EODs, or after administration of certain drugs, chirp production may increase one-thousand-fold to rates as high as 2 s^-1^ (Zupanc and Maler 1993; Engler and Zupanc 2001; Eske et al. 2023).

Chirps last between some tens and a few hundred milliseconds and involve complex frequency and amplitude modulations. Six distinct chirp types have been identified (Engler et al. 2000; Zupanc et al. 2006). They are defined by differences in duration, extent of the frequency and amplitude modulations, as well as additional features, such as the presence or absence of an undershoot before the frequency returns to baseline levels as evident in time-frequency plots. The usefulness of these features for differentiating different chirp types has been shown in several other studies (Ho et al. 2013a, b; Turner et al. 2007; Oboti et al. 2023). Most notably, by employing this approach, a comparative analysis revealed an enormous diversity of chirp signals in 13 species of apteronotids, which included not only variation across species but also between congeners and populations of the same species (Turner et al. 2007).

In *A. leptorhynchus*, spontaneously produced chirps are predominantly of type 1, whereas most chirps evoked by the EODs of a neighboring fish (or mimics of such electric signals) or by proper pharmacological stimulation belong to the type 2 category (Engler et al. 2000; Zupanc et al. 2006; Eske et al. 2023). Both type 1 and type 2 chirps are rather short (duration approximately 20 ms) but distinct in terms of the degree of frequency increase (400 Hz versus 100 Hz) and amplitude reduction (approximately 50% versus <10%). Longer chirps of type 3–6 are, most typically, generated by older individuals and directed to fish of the other sex.

While chirps can be elicited from either sex, at similar rates, through application of pharmacological agents (Eske et al. 2023), during electric interaction with conspecifics or in response to electric stimuli mimicking a fish’s EOD males chirp at much higher rates than females (Zupanc and Maler 1993; Dulka and Maler 1994; Dunlap et al. 1998; Dunlap 2002; Triefenbach and Zakon 2003; Hupé and Lewis 2008). In addition, chirps are optimally evoked by electric stimuli with frequencies within ±10 Hz of the fish’s EOD frequency (Engler and Zupanc 2001). Thus, type 2 chirps are typically exchanged by males. Moreover, the chirps produced by two electrically interacting fish are not independent of each other (Zupanc et al. 2006). Instead, the chirps generated by one fish follow the chirps of the other individual with a preferred latency of roughly 500–1000 ms (Zupanc et al. 2006). This ‘echo response’ may serve a communicatory function during social interactions, such as aggressive encounters.

Traditionally, different chirp types have been identified and quantified by visual inspection of time–voltage and time–frequency plots (e.g., Engler et al. 2000; Engler and Zupanc 2001; Zupanc et al. 2001; Dunlap and Larkins-Ford 2003; Zupanc et al. 2006; Kolodziejski et al. 2007; Hupé and Lewis 2008; Smith and Combs 2008; Dunlap et al. 2011; Gama Salgado and Zupanc 2011; Neeley et al. 2018). In addition, threshold-based algorithms (Bastian et al. 2001; Aumentado-Armstrong et al. 2015; Henninger et al. 2018; Allen and Marsat 2019; Field et al. 2019) and a method based on assumed chirp waveform (Eske et al. 2023) have been used for chirp detection. Whereas these approaches can be successfully employed for the identification of pre-defined chirp types, the definition of chirp categories is subject to the investigator’s bias. Moreover, such approaches do not allow detection of possible additional chirp types that remained unnoticed previously.

To address these deficiencies, we have, in the present study, developed a supervised learning algorithm. Supervised learning is a machine learning paradigm (Bishop 2006) used across many disciplines. Its goal is to learn, from a “ground truth” (GT) data set, a function that assigns proper outputs (in the present study: time instances of chirps and associated chirp types) to inputs (in the present study: time-series frequency and amplitude data). While we demonstrate the suitability of this machine learning paradigm for the unbiased analysis of chirps produced by *A. leptorhynchus*, we propose that similar approaches can be successfully applied to signal analysis in a variety of other ethological and neuroethological systems.

## Materials and methods

### EOD recording

For the present investigation, time–voltage recordings of the EOD containing chirps generated spontaneously or evoked pharmacologically were analyzed. These data had been collected as part of a previous study examining the effect of urethane anesthesia on EOD frequency and chirping behavior in *A. leptorhynchus* (Eske et al. 2023).

Eight fish (total lengths: median, 116 mm; range 107–143 mm; body weights: median, 2.9 g; range 2.5–4.8 g) were used. Their EOD baseline frequencies varied between 683 Hz and 868 Hz (normalized to frequency values expected at 26 ^∘^C, using a Q_10_ of 1.56). The morphological data and EOD frequencies indicate that the fish were approximately 1 year old and included both males and females (Ilieş et al. 2014; Zupanc et al. 2014).

Details of the experiments and the recording technique are given in Eske et al. (2023). Briefly, each fish was kept in an isolation tank in which a cylindrical plastic tube provided shelter. Differential recording of the fish’s EOD was done through a pair of stainless-steel electrodes mounted on the inside of the tube. During recording, the two open ends of the tube were closed with a coarse plastic mesh netting to ensure that the fish did not leave the tube.

The EOD of each fish was recorded for 30 min before, and 180 min immediately after, general anesthesia. State of anesthesia was induced by transferring the fish into a glass beaker containing 2.5% urethane dissolved in water from the fish’s isolation tank. During the pre-anesthesia session, spontaneous chirps occurred but at very low rates of approximately 1 chirp/30 min. Anesthesia induced a tremendous increase in chirping behavior, resulting, on average, in 1500 chirps during the 30 min immediately following anesthesia.

For the present analysis, the 30-min-pre-anesthesia recordings, and the 180-min-post-anesthesia recordings, of the 8 fish were combined, yielding a total of 1680 min of EOD recording. Employing the supervised learning algorithm, a total of 30,734 chirps were detected in these combined recordings.

### Calculation of EOD frequency and amplitude

The sampled voltage data $\left( t_i, v_i\right)$, $i=1, \ldots , M_\textrm{v}$, were exported from Spike 2 and processed in MATLAB version R2021b. These data were filtered in 3-s windows with 2-s overlap using a bandpass filter with frequency band $[0.5, 1.5]\times f_0$, where the fundamental frequency $f_0$ in each 3-s window was determined based on the power spectrum of the signal using fast Fourier transform and the “findpeaks” function of MATLAB.

**Fig. 1:**
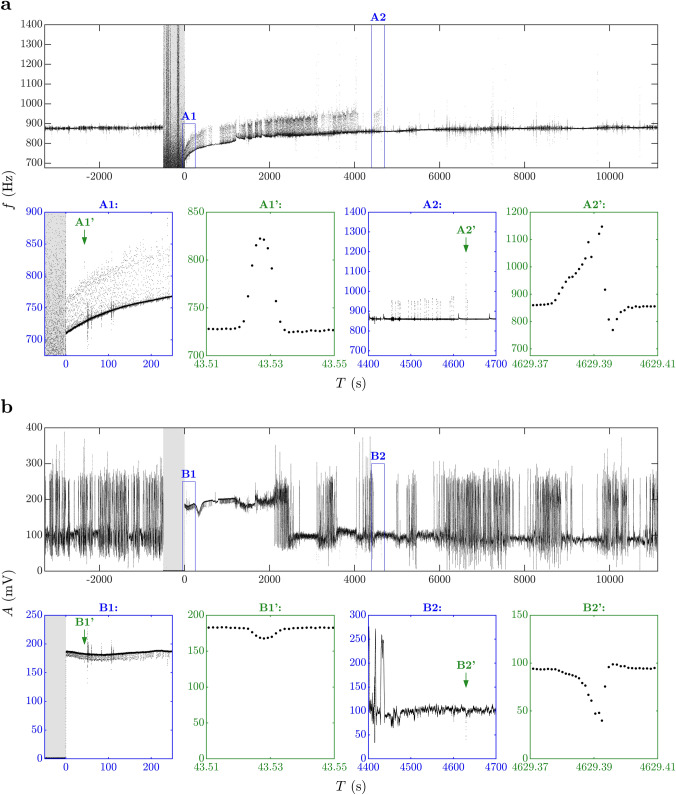
EOD frequency *f* (**a**) and amplitude *A* (**b**) with respect to time *T* in a recording involving urethane anesthesia (for details of computation see Sect. “Calculation of EOD frequency and amplitude”). After baseline recording, the tube with the fish was transferred from the home tank to a glass beaker containing 2.5% urethane solution dissolved in aquarium water. As soon as the fish stopped undulating its anal fin and moving its opercula, it was returned to the home tank (arbitrarily defined as time point $T=0$). The gray bar indicates the time during which the fish was exposed to the anesthetic. Changes in the orientation and position of the fish relative to the recording electrodes result in noisy amplitude signals (pre-anesthesia, and $T>\sim 2000$ s as shown in **b**). The reduction of noise immediately after anesthesia is related to the ceased movement of the fish. Note onset of type 2 chirping at higher rates immediately after anesthesia (**a**/A1, **b**/B1) that persists to approximately $T=4600$ s after exposure to the anesthetic (**a**/A2, **b**/B2). The recorded signal contains both type 2 (**a**/A1’, **b**/B1’) and type 1 (**a**/A2’, **b**/B2’) chirps. The latter is characterized by large rise and negative undershoot in frequency (**a**/A2’), as well as a large drop in amplitude (**b**/B2’). By contrast, the former is characterized by a smaller rise without undershoot in frequency (**a**/A1’) and a smaller reduction in amplitude (**b**/B1’)

Based on the zero-crossings of the filtered signal, we then computed the time, frequency, and amplitude values $\left( T_k, f_k, A_k\right)$ associated with each $k=1, \ldots , M,$ oscillation interval (for details, see Appendix A). An example of computed time-series data of frequency and amplitude is shown in Fig. 1.

### Chirp detection by supervised learning

#### “Ground Truth” data set

*Data collection*

Tuples of equal-time-length time-series data segments

1 $$\begin{aligned} \textbf{S}_{n_\textrm{s}(r-1)+j} \!= & {} \!\left( \!\left\{ T_k^{(r)}, f_k^{(r)}, A_k^{(r)}\right\} : T_k^{(r)}\!\in \!\left[ T_\textrm{start} + (j-1)\Delta T, T_\textrm{start} + j\Delta T\right] ,\right. \nonumber \\{} & {} \left. k=1,\ldots , M-1 \right) , \quad j=1, \ldots , n_\textrm{s}, \end{aligned}$$

were collected from each recording $r=1, \ldots , n_\textrm{r}$, where $n_\textrm{r}$ is the total number of EOD recordings, and superscript $\square ^{(r)}$ indicates association with recording *r*. The time length of segments was determined as $\Delta T = \left( T_\textrm{end}-T_\textrm{start}\right) \!/n_\textrm{s}$. The values of parameters $T_\textrm{start}, T_\textrm{end}, n_\textrm{s}, n_\textrm{r}$, used for the generation of time-series data segments are provided in Table 1.

**Fig. 2:**
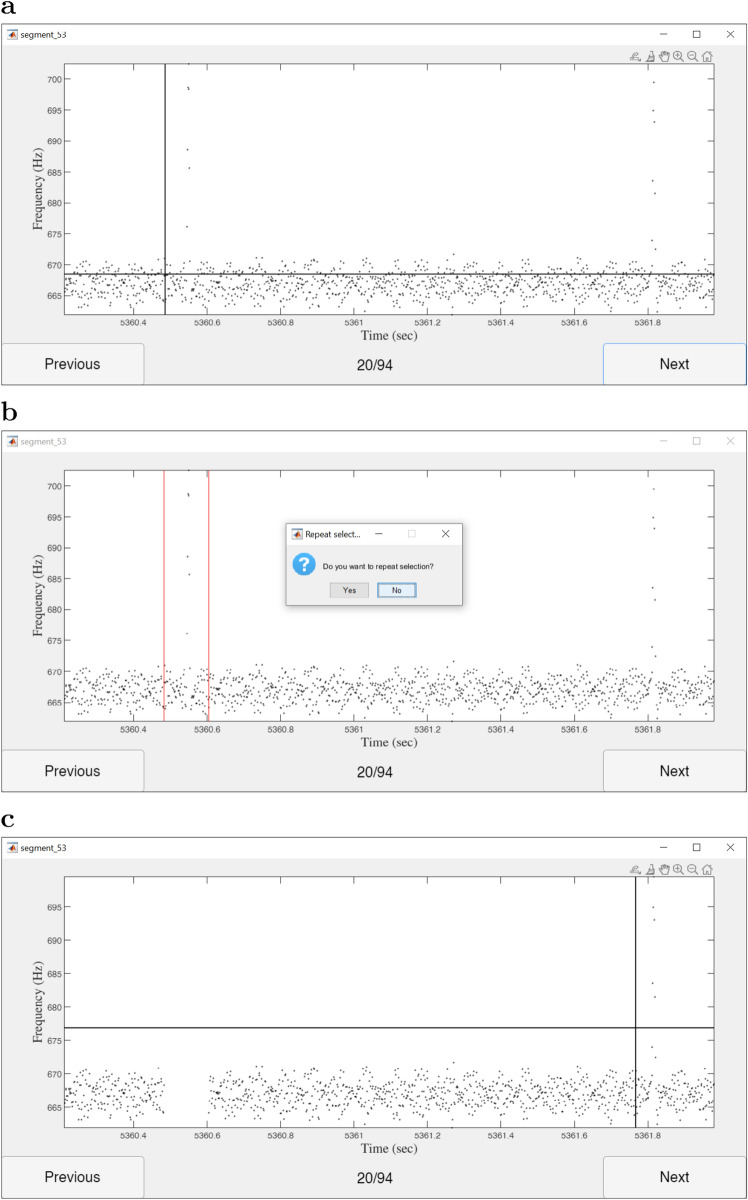
Matlab tool built for collecting chirp samples from time-series frequency data (black dots). The user can select data points associated with a chirp by moving the cursor (intersection of *black lines* in **a** and **c**) to the two end points of the time interval delimiting the chirp instance. After selecting the time interval (red lines in **b**), the user must confirm the current selection before proceeding to collect further data points (see dialog box in **b**). Following the confirmation of the selection, data points associated with the selected time interval are stored and removed from the displayed data set (**c**). Once all displayed chirp instances have been collected, the user can move to the next (or previous), overlapping, time segment to collect the remaining chirp data points from the time-series frequency data segment

Using the MATLAB tool shown in Fig. 2, a person previously trained to identify chirps collected all chirp instances from each segment $\textbf{S}_i$ for all indices $i\in \textbf{i}_\textrm{GT}$, where the elements of subset $\textbf{i}_{\textrm{GT}}\subset \left\{ 1, \ldots , n_\textrm{s}n_\textrm{r}\right\}$, with $n_\textrm{GT}=\left| \textbf{i}_{\textrm{GT}}\right|$ (see Table 1), were randomly chosen, without replacement.

Although for each data point only time and frequency values were displayed during data collection (see Fig. 2), the associated amplitude values were also stored in the GT set of chirps

2 $$\begin{aligned} \textbf{G} = \left\{ \left( \left\{ T_{i, j}, f_{i, j}, A_{i, j}\right\} \right) _{j=1}^{l_i}\right\} _{i=1}^{n}, \end{aligned}$$

where $\left\{ T_{i, j}, f_{i, j}, A_{i, j}\right\}$ is the *j*-th data point of the *i*-th GT chirp sample, $l_i$ denotes the number of data points in the *i*-th sample, and *n* is the total number of samples.

**Table 1:** Parameter values used for generating time-series data segments $\textbf{S}_i$, $i\in \textbf{i}_\textrm{GT}$, from which “ground truth” chirp samples $\textbf{G}$ were collected

Parameter (unit)	Value
$T_\textrm{start}$ (s)	3200
$T_\textrm{end}$ (s)	6200
$n_\textrm{s}$ (1)	10
$n_\textrm{r}$ (1)	16
$n_\textrm{GT}$ (1)	64

Values of $T_\textrm{start}$ and $T_\textrm{end}$ were measured in time coordinates fixed to the start of recordings

*Data processing*

The person who collected chirp samples was instructed to include, in each sample, data points prior to and after chirping, associated with the non-modulated, instantaneous “base” frequency of the fish. Hence, we assumed that each sample includes both pre and post-chirp data points and estimated the “base” frequency and amplitude of each sample *i* as

3 $$\begin{aligned} f_{\textrm{base}, i}&= \textrm{median}\left( \left\{ f_{i, j}\right\} _{j=1}^{n_\textrm{med}}, \left\{ f_{i, l_i-j+1}\right\} _{j=1}^{n_\textrm{med}}\right) , \end{aligned}$$

4 $$\begin{aligned} A_{\textrm{base}, i}&= \textrm{median}\left( \left\{ A_{i, j}\right\} _{j=1}^{n_\textrm{med}}, \left\{ A_{i, l_i-j+1}\right\} _{j=1}^{n_\textrm{med}}\right) , \end{aligned}$$

where $n_\textrm{med} < \underset{i}{\min }(l_i/2)$ is an arbitrarily chosen positive integer which we set to $n_\textrm{med}=10$. We normalized each sample $i=1, \ldots , n$ with respect to the maximum frequency rise according to

5 $$\begin{aligned} \varphi _{i, j} = \frac{f_{i, j} - f_{\textrm{base}, i}}{\underset{j\in \left\{ 1, \ldots , l_i\right\} }{\max }\!\left( f_{i, j}\right) - f_{\textrm{base}, i}}, \quad j=1, \ldots , l_i , \end{aligned}$$

and with respect to the base amplitude as

6 $$\begin{aligned} a_{i, j} = \frac{A_{i, j} - A_{\textrm{base}, i}}{A_{\textrm{base}, i}}, \quad j=1, \ldots , l_i . \end{aligned}$$

Then, we centered the time values of each sample according to

7 $$\begin{aligned}{} & {} \tilde{T}_{i, j}:= T_{i, j} - T_{i, j_{\textrm{cen}, i}}, \quad j=1, \ldots , l_i, \end{aligned}$$

8 $$\begin{aligned}{} & {} j_{\textrm{cen}, i} = \underset{k}{\textrm{argmin}}\left( \left| H_{i, k}-\frac{1}{2}\right| \right) , \end{aligned}$$

9 $$\begin{aligned}{} & {} H_{i, k} = \frac{\sum _{j=1}^{k}h\!\left( \varphi _{i, j}\right) }{\sum _{j=1}^{l_i}h\!\left( \varphi _{i, j}\right) }, \quad k=1, \ldots , l_i, \end{aligned}$$

where rectifier

10 $$\begin{aligned} h\!\left( \varphi _{i, j}\right) = \frac{\textrm{ln}\!\left( 1+e^{\delta (\vert \varphi _{i, j}\vert -\bar{\varphi }_{i})}\right) }{\delta -\delta \bar{\varphi }_{i}}, \end{aligned}$$

with

11 $$\begin{aligned} \bar{\varphi }_{i}=4\max \!\left( \textrm{sd}\left( \left\{ \varphi _{i, k}\right\} _{k=1}^{n_\textrm{med}}\right) \!,\, \textrm{sd}\left( \left\{ \varphi _{i, l_i-k+1}\right\} _{k=1}^{n_\textrm{med}}\right) \right) , \end{aligned}$$

was applied for the elimination of noise and to highlight “meaningful” parts of the frequency sample. Here $\textrm{sd}\!\left( \cdot \right)$ denotes the standard deviation, $\bar{\varphi }_{i}$ is the cutoff value of normalized frequency associated with sample *i* and $\delta =50$ is an arbitrarily chosen smoothing parameter.

Using the empirical cumulative distribution $H_{i, \cdot }$ of rectified frequency values $h\!\left( \varphi _{i, \cdot }\right)$, we trimmed each sample, such that only the data points *j* within interval $\tilde{T}_{i, j}\in \left[ -3\Delta \tilde{T}_i, 3\Delta \tilde{T}_i\right]$ were kept, with

12 $$\begin{aligned}{} & {} \Delta \tilde{T}_i = \tilde{T}_{i, j^{+}_i} - \tilde{T}_{i, j^{-}_i}, \end{aligned}$$

13 $$\begin{aligned}{} & {} j^{+}_i =\!\underset{k\in \left\{ 1, \ldots , l_i\right\} }{\textrm{argmin}}\!\left( \left| H_{i, k}-0.9\right| \right) ,\nonumber \\{} & {}  j^{-}_i =\!\underset{k\in \left\{ 1, \ldots , l_i\right\} }{\textrm{argmin}}\!\left( \left| H_{i, k}-0.1\right| \right) . \end{aligned}$$

**Fig. 3:**
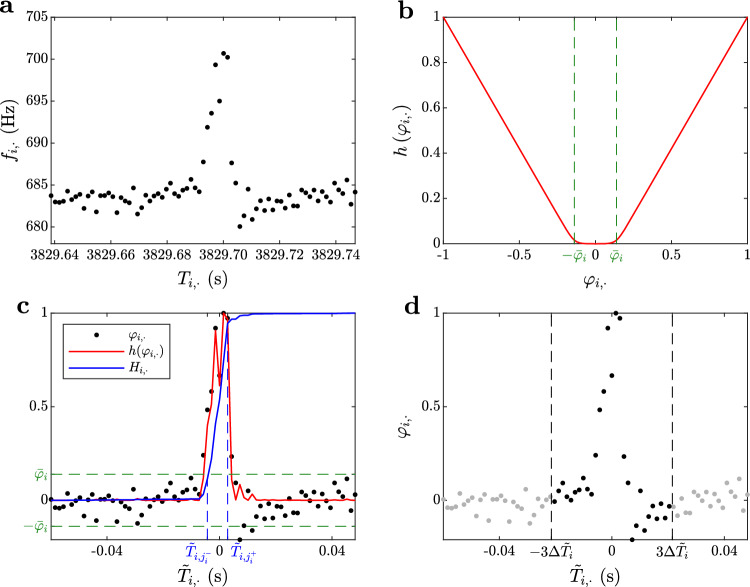
Processing of “ground truth” samples (see Sect. ““Ground Truth” data set”). Data points $\left\{ \left( T_{i,j}, f_{i,j}\right) \right\} _{j=1}^{l_i}$ of the *i*-th sample are plotted in **a** as *black dots*. The frequency values $\left\{ f_{i, j}\right\} _{j=1}^{l_i}$ are normalized according to Eq. 5 and passed through the rectifier function (red curve) displayed in **b**. The green dashed lines in **b** and **c** display the cutoff value $\bar{\varphi }_i$ of the rectifier function. The centered and normalized data points $\left\{ \left( \tilde{T}_{i,j}, \varphi _{i,j}\right) \right\} _{j=1}^{l_i}$ of the *i*-th sample (see Eqs. 5–11) are displayed in **c** as black dots together with the rectified normalized frequencies (red curve) and their empirical cumulative distribution (blue curve). The 10% and 90% percentile estimates (blue, dashed lines in **c**) of this cumulative distribution determine the time width of the sample: $\Delta \tilde{T}_i = \tilde{T}_{i, j_i^+}-\tilde{T}_{i, j_i^-}$. The sample is trimmed based on this time width (**d**) such that data points outside interval $\tilde{T}_{i, j}\in \left[ -3\Delta \tilde{T}_i, 3\Delta \tilde{T}_i\right]$ (delimited by black, dashed lines and marked by *gray dots*) are eliminated

Note that here $\Delta \tilde{T}_i$ is the difference between the 90% and 10% percentile estimates of the empirical cumulative distribution $H_{i, \cdot }$. The above described data processing method is illustrated in Fig. 3.

*Grouping and resampling*

Because our supervised learning method requires uniform size among GT samples, we grouped and resampled all GT samples according to the number of data points that formed the individual GT samples.

After trimming, the size of each GT sample was roughly commensurate with the length of the associated chirp. To distinguish between chirps whose duration have different time scales, we divided GT samples into three groups and resampled the members of each *r* group such that associated samples contained $10^r+1$ number of points:

14 $$\begin{aligned} \textbf{G}_r= & {} \left\{ \left\{ \left( T_{i, j_{\textrm{cen}, i}+j}, f_{i, j_{\textrm{cen}, i}+j}, A_{i, j_{\textrm{cen}, i}+j}\right) \right\} _{j=-10^r/2}^{10^r/2}: \right. \nonumber \\{} & {} \quad \left. \left| \left\{ \tilde{T}_{i, j}: \tilde{T}_{i, j}\!\in \!\!\left[ -3\Delta \tilde{T}_i, 3\Delta \tilde{T}_i\right] \!, 1\le j\le l_i\right\} \right| \! \in \!\left( 10^{r-1}\!+\!1, 10^{r}\!+\!1\right] \!,\right. \nonumber \\{} & {} \quad \left. 1\le i\le n\right\} , \quad r=1, 2, 3. \end{aligned}$$

Here we utilized the fact that all data points inside any GT sample can be located within the associated recording’s time-frequency-amplitude data. For example, if we know that $T_{i, 1}$ and $T_q$ are from the same recording and that $T_{i, 1} = T_q$, then we can find any other point *j* associated with sample *i*: $\left( T_{i, j}, f_{i, j}, A_{i, j}\right) = \left( T_{q+j-1}, f_{q+j-1}, A_{q+j-1}\right)$.

Note that chirps typically have a duration shorter than 0.5 s, and the highest EOD frequency in *A. leptorhynchus* is approximately 1000 Hz, therefore GT sample groups $\textbf{G}_r$, $r=1, 2, 3,$ are able to capture the full length of all chirps.

#### Training

*Principal component analysis*

After resampling, we recomputed, according to Eqs. 3–6, the normalized frequencies and amplitudes $\left( \varphi _{i, j_{\textrm{cen}, i}+j}, a_{i, j_{\textrm{cen}, i}+j}\right) , j=-10^r/2, \ldots , 10^r/2$, of each chirp sample *i* in each GT group $\textbf{G}_r$. For ease of notation, in the following, we drop the shift $j_{\textrm{cen}, i}$ in the second subscript index.

For each *r*, we collected from $\textbf{G}_r$ the normalized frequency and amplitude values

15 $$\begin{aligned} \textbf{f}_r^{(i)}&= \left[ \varphi _{i, -10^r/2}, \ldots , \varphi _{i, 10^r/2},\right] ^\textrm{T}, \end{aligned}$$

16 $$\begin{aligned} \textbf{a}_r^{(i)}&= \left[ a_{i, -10^r/2}, \ldots , a_{i, 10^r/2}\right] ^\textrm{T}, \end{aligned}$$

of each sample *i* associated with the training set (for details about the training set, see Sect. “Cross-validation”) into a matrix $\textbf{X}_r\in {\mathbb {R}}^{m_r\times 2\left( 10^r+1\right) }$ such that

17 $$\begin{aligned} \textbf{X}_r^\textrm{T} = \left[ \begin{array}{ccc} \textbf{f}_r^{(1)} &{} \cdots &{} \textbf{f}_r^{(m_r)}\\ \textbf{a}_r^{(1)} &{} \cdots &{} \textbf{a}_r^{(m_r)} \end{array} \right] , \end{aligned}$$

where $m_r$ is the total number of samples in $\textbf{G}_r$ associated with the training set. For the further ease of notation, in the following, we drop index *r*, as well.

We determined the principal components (PCs) $\textbf{p}_1, \ldots , \textbf{p}_{2(10^r+1)},$ of $\textbf{X}$ by performing the spectral decomposition of $\textbf{X}^\textrm{T}\textbf{X}$. Then we projected the training data set onto the space of the first *N* PCs, i.e., we computed

18 $$\begin{aligned} \textbf{Y} = \textbf{X}\textbf{P}_{N}, \end{aligned}$$

where $$\textbf{P}_N=\left[ \textbf{p}_1, \ldots , \textbf{p}_N\right] $$.

*Gaussian mixture model fitting*

We modeled the projected data $\textbf{Y}^\textrm{T}=\left[ \textbf{y}^{(1)}, \ldots , \textbf{y}^{(m)}\right]$ using the Gaussian mixture model (GMM)

19 $$\begin{aligned} \textbf{y}^{(i)} \sim {\mathcal {N}}\left( \varvec{\mu }_c, \varvec{\Sigma }_c\right) , \quad c\sim M_C\left( p_1, \ldots , p_C\right) , \end{aligned}$$

where ${\mathcal {N}}\left( \varvec{\mu }_c, \varvec{\Sigma }_c\right)$ is the multivariate normal distribution of the *c*-th mixture component with mean $\varvec{\mu }_c\in {\mathbb {R}}^{N\times 1}$ and covariance $\varvec{\Sigma }_c\in {\mathbb {R}}^{N\times N}$, while $M_C\left( p_1, \ldots , p_C\right)$ is a multinomial distribution with *C* number of categories and mixing proportions $p_1, \ldots , p_C$. We estimated the unknown parameters $\varvec{\Theta }=\left\{ p_1, \ldots , p_C, \varvec{\mu }_1, \ldots , \varvec{\mu }_C, \varvec{\Sigma }_1, \ldots , \varvec{\Sigma }_C\right\}$ of this GMM based on data $\textbf{Y}$ using the “fitgmdist” function of MATLAB.

*Elimination of outliers*

After fitting the GMM, we assigned each data sample *i* to the cluster with maximum posterior probability, i.e., we computed the cluster of sample *i* according to

20 $$\begin{aligned} c_i = \underset{c\in \left\{ 1, \ldots , C\right\} }{\textrm{argmax}}\!\left( P\left( c\vert i\right) \right) , \end{aligned}$$

for each $i=1, \ldots , m$, where $P\!\left( c\vert i\right)$ is the probability that sample *i* belongs to cluster *c*, given the observation $\textbf{y}^{(i)}$. Then, we computed the coefficient of determination (CoD) of the frequency component of each sample with respect to its assigned cluster mean as

21 $$\begin{aligned} R^{2}_{i} = 1 - \frac{\left\| \textbf{f}^{(i)}-\bar{\textbf{f}}_{c_i}\right\| ^2}{\left\| \textbf{f}^{(i)}-\bar{\textbf{f}}^{(i)}\right\| ^2}. \end{aligned}$$

Here $\left\| \cdot \right\|$ denotes the L2 norm and

22 $$\begin{aligned}{} & {} \left[ \bar{\textbf{f}}_c, \bar{\textbf{a}}_c\right] ^\textrm{T} = \textbf{P}_N\hat{\varvec{\mu }}_c , \end{aligned}$$

23 $$\begin{aligned}{} & {} \bar{\textbf{f}}^{(i)}=\frac{1}{10^r+1}\left( \textbf{1}^\textrm{T}\textbf{f}^{(i)}\right) \textbf{1} , \end{aligned}$$

with $\textbf{1}$ being a vector of 1-s.

We eliminated each cluster *c* for which the 5% percentile of associated CoD values $\left\{ R^{2}_{i}: c_i=c, 1\le i\le m\right\}$ was below threshold $\delta _{R^2}=0.3$. Additionally, we eliminated each cluster *c* whose size $\left| \left\{ i:c_i=c, 1\le i\le m\right\} \right|$ was below threshold $\delta _c=30$.

**Fig. 4:**
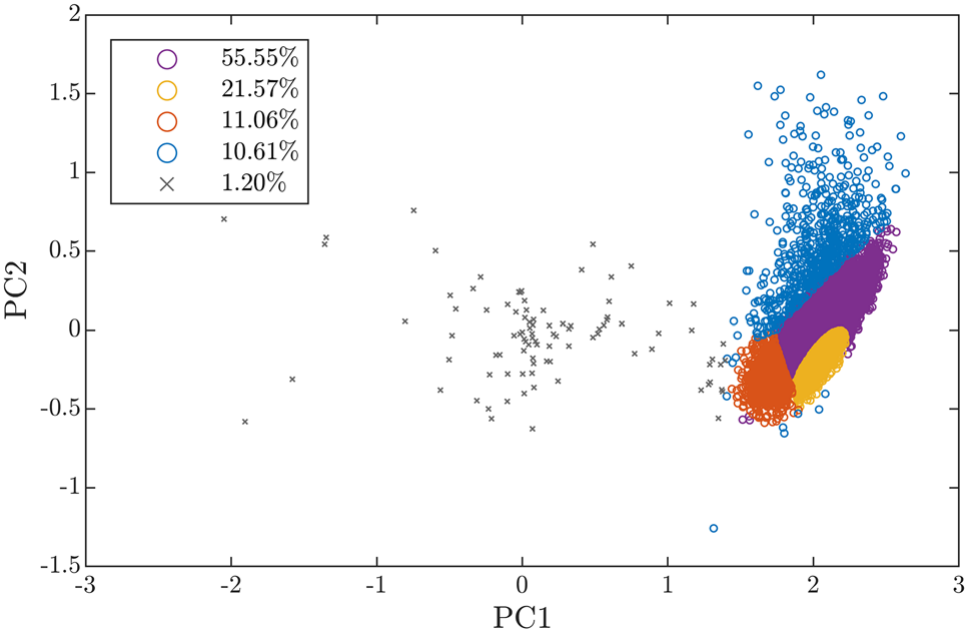
Training data set projected to the space spanned by the first two principal components (PC1 and PC2). Circles with different colors correspond to clusters identified by the algorithm. Gray crosses correspond to samples in an eliminated cluster. The percentage-wise size of kept (circles) and eliminated (crosses) clusters is indicated at the top left corner, relative to the size of the training set

Figure 4 illustrates the projected training data $\textbf{Y}$ from $\textbf{G}_2$, with parameters $N=2$ and $C=5$; note the eliminated cluster.

#### Detection

Training yields PCs $\textbf{P}_N$ and GMM

24 $$\begin{aligned} \textbf{y}^{(i)} \sim {\mathcal {N}}\left( \hat{\varvec{\mu }}_c, \hat{\varvec{\Sigma }}_c\right) , \quad c\sim M_{C^*}\!\left( \tilde{p}_1, \ldots , \tilde{p}_{C^*}\right) , \end{aligned}$$

where $C^*\le C$ is the number of kept clusters, with $\tilde{p}_c=\hat{p}_c/\sum _{q=1}^{C^*}\hat{p}_q$, and $\hat{p}_c,\hat{\varvec{\mu }}_c, \hat{\varvec{\Sigma }}_c$, being the estimated parameters of kept clusters $c=1, \ldots , C^*$.

**Fig. 5:**
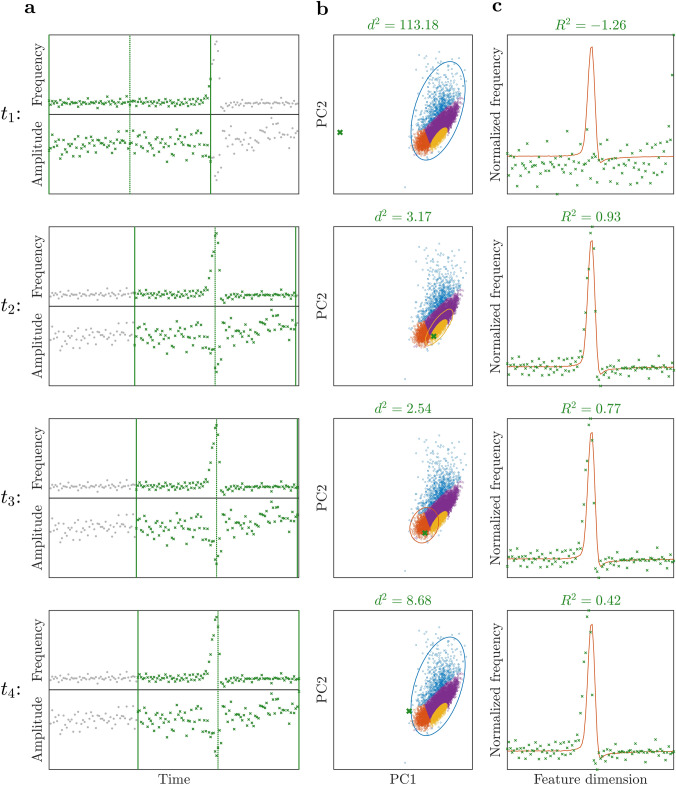
Illustration of the chirp detection methods described in Sect. “Detection”. Different rows correspond to different time instants ($t_1<t_2<t_3<t_4$) of the “sliding” time window indicated by vertical *green lines* in **a**. At each time instant, the Mahalanobis-distance-based detection algorithm (**b**) normalizes the data set inside the time window (green crosses in **a**) and projects it to the space spanned by the principal components of the training set (green cross in **b**). If the squared Mahalanobis distance value $d^2$ associated with this projected point (indicated at the top of each row in **b**) is below the limit of the cluster with highest posterior probability (corresponding to the color-coded ellipse in **b**), then the Mahalanobis-distance-based algorithm may detect a chirp (2nd and 3rd row). At each time instant, the coefficient-of-determination-based algorithm (**c**) normalizes the data set inside the time window and computes its coefficient of determination with respect to each cluster mean. The highest coefficient-of-determination value $R^2$ among all cluster means is indicated at the top of each row in **c**, and the related cluster mean is plotted (color-coded line in **c**). If this value is above a threshold, then the coefficient-of-determination-based algorithm may detect a chirp

To detect chirps in recordings, we analyzed data points $\left\{ \left( T_{i+j-1}, f_{i+j-1}, A_{i+j-1} \right) \right\}_{j=1}^{10^r+1}$, $i=1, \ldots , M-10^r$ in a moving time window containing $10^r+1$ samples (see Fig. 5a). At each instance *i*, we computed normalized frequency and amplitude values

25 $$\begin{aligned} \textbf{f}^{(i)}&= \left[ \varphi _{i, 1}, \ldots , \varphi _{i, 10^r+1},\right] ^\textrm{T}, \end{aligned}$$

26 $$\begin{aligned} \textbf{a}^{(i)}&= \left[ a_{i, 1}, \ldots , a_{i, 10^r+1}\right] ^\textrm{T}, \end{aligned}$$

according to formulas Eqs. 3–6 with $\left( T_{i, j}, f_{i, j}, A_{i, j}\right) = \left( T_{i+j-1}, f_{i+j-1}, A_{i+j-1}\right)$ and $l_i = 10^r+1$.

*Mahalanobis-distance-based detection*

At each instance *i*, our Mahalanobis-distance-based (MDB) detection method first projects the normalized frequency and amplitude data onto the PCs according to

27 $$\begin{aligned} \textbf{y}^{(i)} = \textbf{P}_N^\textrm{T} \left[ \begin{array}{c} \textbf{f}^{(i)}\\ \textbf{a}^{(i)} \end{array}\right] , \end{aligned}$$

then it determines the kept cluster which is most likely to generate $\textbf{y}^{(i)}$:

28 $$\begin{aligned} c_i = \underset{j\in \left\{ 1, \ldots , C^*\right\} }{\textrm{argmax}}\!\left( P\!\left( j\vert i\right) \right) . \end{aligned}$$

Afterward, our method computes the Mahalanobis distance

29 $$\begin{aligned} d_i = \sqrt{\left( \textbf{y}^{(i)}-\hat{\varvec{\mu }}_{c_i}\right) ^\textrm{T} \hat{\varvec{\Sigma }}_{c_i}^{-1}\left( \textbf{y}^{(i)}-\hat{\varvec{\mu }}_{c_i}\right) }. \end{aligned}$$

For any point generated by kept cluster $c_i$, realizations $d_i^2$ follow a chi-squared distribution with *N* degrees of freedom: $D_i^2\sim \chi ^2_N$.

The MDB method collects all *i* instances, where the squared Mahalanobis distance is below threshold $\varepsilon _{d^2}$ and the maximum frequency rise is above threshold $\varepsilon _{f}$, into the tuple

30 $$\begin{aligned} \textbf{c}_{\textrm{MDB}}= & {} \left( i: d_i^2<\varepsilon _{d^2}, \underset{1\le j\le 10^r+1}{\max }\!\left( f_{i, j}\right) -f_{\textrm{base}, i}>\varepsilon _{f},\right. \nonumber \\{} & {} \quad \left. \left. i=1, \ldots , M - 10^r - 1 \right. \right) . \end{aligned}$$

Each contiguous segment in $\textbf{c}_{\textrm{MDB}}$ corresponds to an identified chirp. In each contiguous segment, we associate the identified chirp with the instance *i* that has lowest distance $d_i$. Threshold $\varepsilon _{d^2}$ is determined based on a chosen level of significance $\alpha$ such that $P\left( D_i^2<\varepsilon _{d^2}\right) =1-\alpha$. The MDB method is illustrated in Fig. 5b.

*Coefficient-of-determination-based detection*

At each instance *i*, our coefficient-of-determination-based (CDB) detection method computes the CoD of the frequency component with respect to each kept cluster mean according to

31 $$\begin{aligned} R^{2}_{i,c} = 1 - \frac{\left\| \textbf{f}^{(i)}-\bar{\textbf{f}}_{c}\right\| ^2}{\left\| \textbf{f}^{(i)}-\bar{\textbf{f}}^{(i)}\right\| ^2}, \quad c=1, \ldots , C^*, \end{aligned}$$

using formulae Eqs. 22–23, and assigns instance *i* to the cluster with highest CoD value:

32 $$\begin{aligned} c_i = \underset{c\in \left\{ 1, \ldots , C^*\right\} }{\textrm{argmax}}\!\left( R^2_{i, c}\right) . \end{aligned}$$

Afterward, the CDB method collects all instances into the tuple $\textbf{c}_{\textrm{CDB}}$ where the CoD value and the maximum frequency rise are both above thresholds $\varepsilon _{R^2}$ and $\varepsilon _{f}$, respectively:

33 $$\begin{aligned} \textbf{c}_{\textrm{CDB}}= & {} \left( i: R^2_{i, c_i}>\varepsilon _{R^2}, \underset{1\le j\le 10^r+1}{\max }\!\left( f_{i, j}\right) -f_{\textrm{base}, i}>\varepsilon _{f},\right. \nonumber \\{} & {} \left. \left. i=1, \ldots , M - 10^r - 1 \right. \right) . \end{aligned}$$

Similarly to the MDB method, identified chirps are associated with contiguous segments in $\textbf{c}_{\textrm{CDB}}$. In each contiguous segment, the identified chirp is assigned to the instance *i* that has the highest $R^2_{i, c_i}$ value. The CDB method is illustrated in Fig. 5c.

### Chirp detection based on assumed chirp waveform

**Fig. 6:**
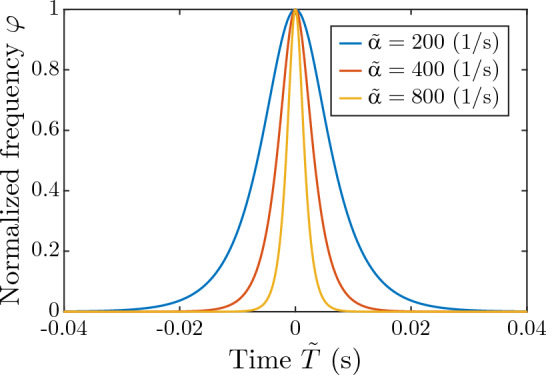
Time–frequency shape function used for chirp detection in Eske et al. (2023). The time course of normalized frequency (see Eq. 5) during chirping is modeled by a single-parameter function $\varphi \!\left( \tilde{T}; \tilde{\alpha} \right)$. Different colors correspond to different shape parameter values $\tilde{\alpha}$

In order to assess the performance of the two algorithms detailed above, we chose, as a reference, the time-frequency-shape-based (TFSB) chirp detection algorithm described in (Eske et al. 2023). This algorithm is based on the chirp waveform function

34 $$\begin{aligned} \varphi \!\left( \tilde{T};\tilde{\alpha }\right) = \dfrac{2e^{\tilde{\alpha } \tilde{T}}}{1+e^{2\tilde{\alpha } \tilde{T}}}, \end{aligned}$$

which is assumed to characterize, during chirps, the normalized frequency $\varphi$ with respect to time $\tilde{T}$. This function is parameterized by a single parameter $\tilde{\alpha }$ that controls chirp duration (see Fig. 6).

The TFSB algorithm has 7 hyper-parameters, out of which we fixed 5 (see Table 2), and the remaining 2 we determined via cross-validation (see Sect. “Cross-validation”).

**Table 2:** Fixed hyper-parameters of the time-frequency-shape-based chirp detection algorithm (for definition of parameters, see Eske et al. 2023)

Parameter (unit)	Value
$n_\textrm{wind}$ (1)	100
$n_\textrm{med}$ (1)	10
$n_\alpha$ (1)	200
$\alpha _\textrm{min}$ (1/s)	100
$\alpha _\textrm{max}$ (1/s)	800

### Cross-validation

To determine the optimal hyper-parameter values $\textbf{h}_\textrm{opt}$ of detection algorithms, we used *k*-fold cross-validation. In particular, we randomized indices $i\in \textbf{i}_\textrm{GT}$ associated with time-series data segments $\textbf{S}_i$ and split them onto *k* number of equal-size folds: $\textbf{i}_{\textrm{GT}, q}\subset \textbf{i}_\textrm{GT}$, $q=1, \ldots , k$. For each iteration step $q=1, \ldots , k,$ of cross validation, a single fold $\textbf{i}_{\textrm{GT}, q}$ was used as a test set for determining the performance of the algorithm, while the rest of the folds were used as a training set. Note that only the two supervised algorithms were trained (for details, see Sect. “Training”), while the TFSB algorithm did not involve any training (Eske et al. 2023). The performance of each algorithm was determined by computing the false positive and false negative rates for each iteration step $q=1, \ldots , k$, as

35 $$\begin{aligned} \textrm{FP}_q&= \!\frac{\sum \limits _{s\in \textbf{i}_{\textrm{GT}\!, q}}\!\sum \limits _{j=1}^{m_{\textrm{A}, s}}\!\mathbbm {1}\!\left( \left| \left\{ i:\hat{T}_j^{(s)}\!\notin \!\left[ T_{i, 1}^{(s)}, T_{i, l_i}^{(s)}\right] \!, 1 \le i \le m_{\textrm{GT},s}\right\} \right| \!\!=\!m_{\textrm{GT}, s}\right) }{\sum \limits _{s\in \textbf{i}_{\textrm{GT}\!, q}}\!\!m_{\textrm{A}, s}}, \end{aligned}$$

36 $$\begin{aligned} \textrm{FN}_q&= 1 - \frac{\sum \limits _{s\in \textbf{i}_{\textrm{GT}\!, q}}\!\!\!\sum \limits _{i=1}^{m_{\textrm{GT}, s}}\!\mathbbm {1}\!\left( \left| \left\{ j:\hat{T}_j^{(s)}\!\in \!\left[ T_{i, 1}^{(s)}, T_{i, l_i}^{(s)}\right] \!, 1 \le j \le m_{\textrm{A},s}\right\} \right| \!>\!0\right) }{\sum \limits _{s\in \textbf{i}_{\textrm{GT}\!, q}}\!\!m_{\textrm{GT}, s}}, \end{aligned}$$

where $\mathbbm {1}\!\left( \cdot \right)$ is the indicator function, $\hat{T}^{(s)}_j$ denotes the *j*-th time instance of chirps detected by the algorithm in time-series data segment $\textbf{S}_s$, while $T_{i, 1}^{(s)}$ and $T_{i, l_i}^{(s)}$ correspond to the first and last data point of the *i*-th chirp sample in $\textbf{G}_r$ collected from data segment $\textbf{S}_s$. Parameters $m_{\textrm{A}, s}$ and $m_{\textrm{GT}, s}$ denote the total number of chirps detected by the algorithm in $\textbf{S}_s$, and collected manually from $\textbf{S}_s$, respectively. The overall performance of the algorithm was determined by averaging over all folds:

37 $$\begin{aligned} \overline{\textrm{FP}}(\textbf{h}) = \frac{1}{k}\sum _{q=1}^{k}\textrm{FP}_q(\textbf{h}), \quad \overline{\textrm{FN}}(\textbf{h}) = \frac{1}{k}\sum _{q=1}^{k}\textrm{FN}_q(\textbf{h}). \end{aligned}$$

Note that false positive and false negative rates depend on hyper-parameters $\textbf{h}$. We tuned the hyper-parameters such that for a given maximum tolerated average false positive rate $r_\textrm{FP}$, the average false negative rate is minimized, i.e.,

38 $$\begin{aligned} \textbf{h}_\textrm{opt}\!\left( r_\textrm{FP}\right) = \underset{\textbf{h}\in \textbf{H}\left( r_\textrm{FP}\right) }{\textrm{argmin}}\!\left( \overline{\textrm{FN}}(\textbf{h})\right) , \quad \textbf{H}(r_\textrm{FP})=\left\{ \textbf{h}\in \mathbf {\Omega }: \overline{\textrm{FP}}(\textbf{h})\le r_\textrm{FP}\right\} , \end{aligned}$$

where $\mathbf {\Omega }$ is the search domain of hyper-parameters. At the maximum tolerated average false positive rate $r_\textrm{FP}$, the lowest achievable average false negative rate is

39 $$\begin{aligned} r_\textrm{FN}\!\left( r_\textrm{FP}\right) = \overline{\textrm{FN}}\!\left( \textbf{h}_\textrm{opt}\!\left( r_\textrm{FP}\right) \right) . \end{aligned}$$

The implemented search domains of hyper-parameters are summarized in Table 3.

**Table 3:** Search domains of hyper-parameters for the Mahalanobis-distance-based (MDB), coefficient-of-determination-based (CDB), and time-frequency-shape-based (TFSB) chirp detection algorithms

	Search domain		
Hyper-parameter	MDB	CDB	TFSB
*N*	$\left\{ 2, 3, \ldots , 8\right\}$	$\left\{ 2, 3, \ldots , 8\right\}$	n/a
*C*	$\left\{ 2, 3, \ldots , 14\right\}$	$\left\{ 2, 3, \ldots , 14\right\}$	n/a
$\varepsilon _{f}$	$\left\{ 0, 2, \ldots , 20\right\}$	$\left\{ 0, 2, \ldots , 20\right\}$	$\left\{ 0, 2, \ldots , 20\right\}$
$\alpha$	$\left\{ 0.01, 0.02, \ldots , 0.15\right\}$	n/a	n/a
$\varepsilon _{R^2}$	n/a	$\left\{ 0.3, 0.35, \ldots , 0.8\right\}$	$\left\{ 0.3, 0.35, \ldots , 0.8\right\}$

## Results

### Performance of detection algorithms

**Fig. 7:**
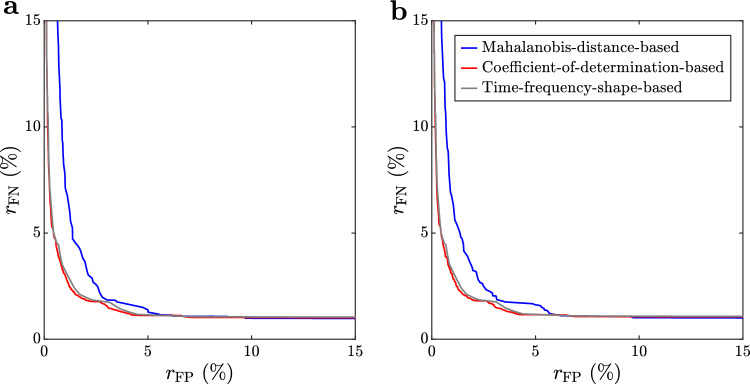
Lowest achievable average false negative rate ($r_\textrm{FN}$) as a function of maximum allowed average false positive rate ($r_\textrm{FP}$), using *k*-fold cross-validation with $k=2$ (**a**) and $k=4$ (**b**). Curves were calculated for “ground truth” data set $\textbf{G}_2$ according to Sect. “Cross-validation”. Different colors are associated with different methods, as indicated in the top right corner of **b**

For the GT group $\textbf{G}_2$, we computed the lowest achievable average false negative rate $r_\textrm{FN}$ of each algorithm at given average false positive rate tolerances $r_\textrm{FP}$ (see Fig. 7) according to Eq. 39, using the search domains in Table 3. These results show that the performance of the MDB method is inferior to the CDB and TFSB methods. The CDB method performs better than the MDB and TFSB methods, although, the $r_\textrm{FN}\!\left( r_\textrm{FP}\right)$ curves of the CDB and TFSB methods are nearly identical (Fig. 7).

### Principal components and explained variance

**Fig. 8:**
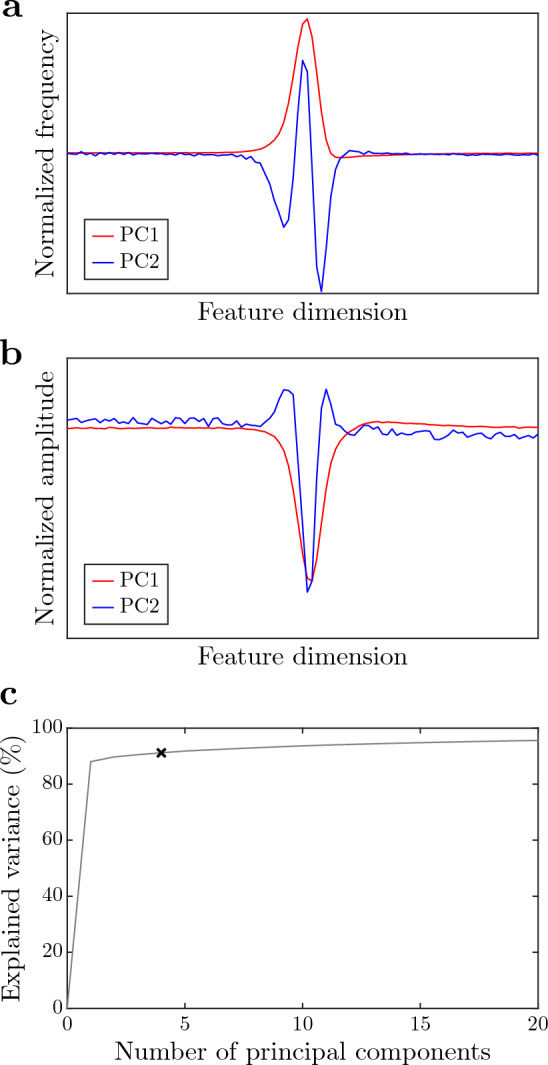
Normalized frequency (**a**) and amplitude (**b**) in the first two principal components (PC1 and PC2) of the “ground truth” data set $\textbf{G}_2$. Explained variance as a function of retained principal components (*gray line* in **c**). The *black cross* in **c** corresponds to optimal hyper-parameter settings $\textbf{h}_\textrm{opt}\!\left( r_\textrm{FP}=5\%\right)$ determined by 4-fold cross-validation (see Sect. “Cross-validation”)

To illustrate waveform components that dominate GT group $\textbf{G}_2$, we computed its PCs (Fig. 8a, b) and the explained variance in terms of the number of its retained PCs (Fig. 8c). The first PC explains 90% of the variation in $\textbf{G}_2$ (Fig. 8c). The frequency shape of the first PC (PC1 in Fig. 8a, b) is similar to the chirp waveform of the TFSB method (cf. Fig. 6). This, together with the high percentage of explained variance associated with the first PC, result in a similar performance of the TFSB method and the CDB method (Fig. 7).

### Chirp detection

**Fig. 9:**
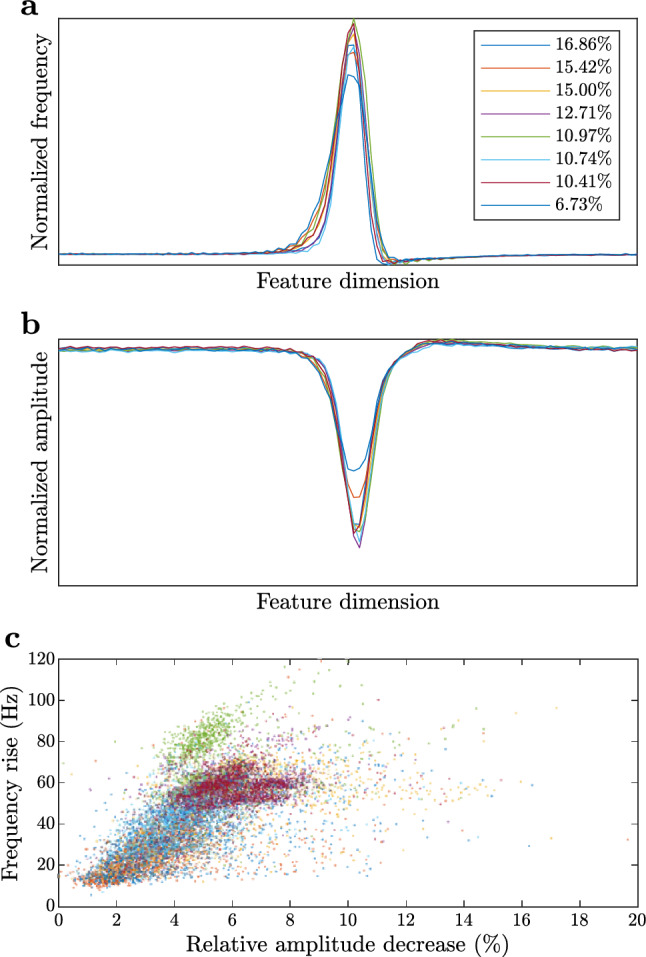
Normalized frequency (**a**) and amplitude (**b**) components of cluster means for the model trained on the entire “ground truth” data set $\textbf{G}_2$. Clusters were computed under optimal parameter settings $\textbf{h}_\textrm{opt}\!\left( r_\textrm{FP}=5\%\right)$ determined via 4-fold cross-validation (see Sect. “Cross-validation”). Relative voltage-amplitude-decrease/maximum-frequency-rise pairs plotted for each sample (**c**) reveal that $\textbf{G}_2$ consists entirely of type 2 chirps. Different colors are associated with different clusters, their proportions are indicated in the top-right corner of **a**

After cross-validation, we trained a model according to Sect. “Training” based on the entire GT data set $\textbf{G}_2$. We used optimal hyper-parameters $\textbf{h}_\textrm{opt}\!\left( r_\textrm{FP}=5\%\right)$ determined via 4-fold cross-validation (see Sect. “Cross-validation”). The cluster means of the model, computed according to Eq. 22, are shown in Fig. 9.

After training, we employed the CDB method (under hyper-parameters $\textbf{h}_\textrm{opt}\!\left( r_\textrm{FP}=5\%\right)$) to detect chirps in all 1680 min of EOD recordings. A total of 30,734 chirps were detected. We further investigated all detected chirps assigned to the cluster mean with the smallest proportion (6.73%, see Fig. 9). To find sub-clusters, we fitted a new GMM on these chirps according to Sect. “Training” using $N=4$ and $C=8$.

**Fig. 10:**
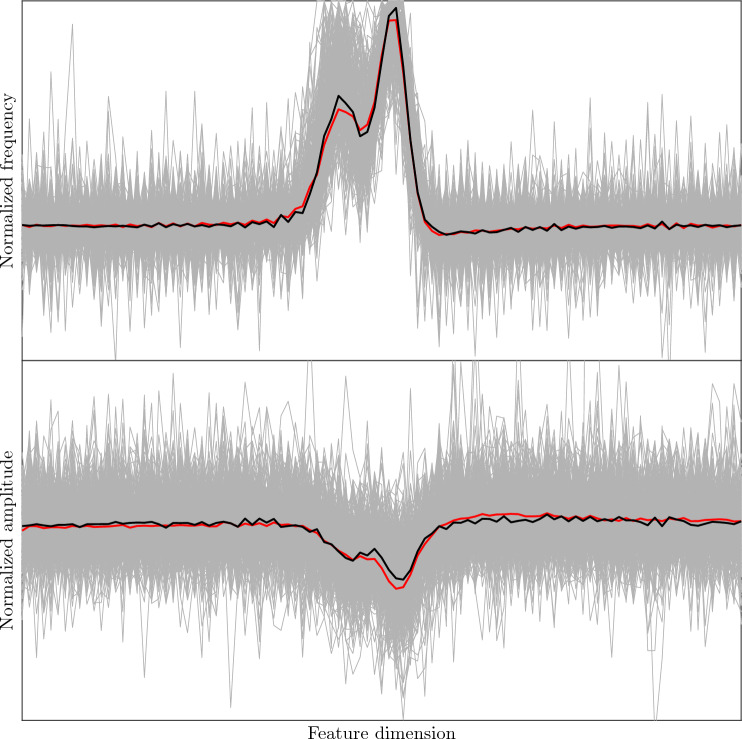
Cluster mean (*red*) and detected chirp samples (gray) in a sub-cluster (containing 264 samples) related to the cluster with 6.73% proportion in Fig. 9a (see Sect. “Chirp detection”). The black curve displays the median of detected chirp samples across feature dimensions

This analysis revealed a new chirp type (see Fig. 10) characterized by short, 20–30 ms duration, and two peaks in frequency rise and amplitude drop. These latter characteristics are distinct from all previously identified chirps of similar duration (c.f. Engler et al. 2000). It is important to note that here we focused on the cluster mean with the smallest proportion. The sub-clustering of chirps assigned to other cluster means may reveal further chirp types.

The distinct feature of this novel type, compared to the previously described six chirp types (Engler et al. 2000; Zupanc et al. 2006), is the existence of two frequency peaks (instead of just one peak); and the occurrence of two amplitude drops—the first, rather modest amplitude decrease is followed by a second, more pronounced reduction. Double frequency peaks have also been found in other apteronotid species, most notably in the *A. bonapartii* group (Turner et al. 2007). However, unlike the duplet frequency modulation characterizing the novel type in *A. leptorhynchus*, in *A. bonapartii* the first frequency increase is followed by a second, less pronounced increase.

## Discussion

### Advantages of the supervised-learning method

The results presented in this paper demonstrate the superiority of our supervised-learning algorithm over traditional methods for analysis of chirps produced by *A. leptorhynchus*.

The first advantage of our method lies in its versatility, compared to traditional approaches. As shown in Sect. “Principal components and explained variance”, the TFSB method performs well for the herein analyzed signal segments because a single time-frequency waveform (associated with type 2 chirps) dominates the collected GT chirp data set, and this waveform matches well the assumed time-frequency shape. If multiple dominant waveforms are present in the GT chirp data set, or if the assumed time-frequency shape does not match the dominant chirp waveform, the performance of the TFSB method would be significantly worse. Furthermore, the design of a shape function representative of the dominant chirp waveform is rather cumbersome and impacted by the researcher’s bias. In contrast, the supervised-learning algorithm autonomously trains chirp waveform models by fitting them to the collected GT chirp data. Given that the GT data set is representative of chirps in the analyzed signal, this algorithm provides an unbiased way for the automatic identification of dominant chirp waveforms in the signal.

The second advantage of our supervised-learning method is its ability to identify, in an unbiased way, possible sub-types of a signal. In the case of chirping behavior in *A. leptorhynchus*, visual inspection of time–frequency plots and time–voltage plots has suggested six subtypes of this signal (Engler et al. 2000; Zupanc et al. 2006). Although, in the present study, the analyzed recordings contained predominantly a single chirp subtype (type 2), our method suggested that further differentiation of this subtype is possible (see Sect. “Chirp detection”).

The third advantage of our method is that, compared to traditional approaches, it extracts more information from the samples used for the validation of the algorithm. Note that only a few traditional approaches validate their algorithm (e.g., Eske et al. 2023) by signals with known chirp types and locations. However, these approaches use the collected set of chirps only to test efficiency, and thus the algorithm itself is not informed by the known chirp content. By contrast, our supervised learning method takes full advantage of known chirps and utilizes them for both training the algorithm and testing its efficiency.

### Limitations of the method

Although our algorithm trains itself and identifies chirp clusters automatically, it still relies on the collection of GT samples. Consequently, results are still impacted by the bias of the individual who collects the chirp samples of the GT set. This bias can be reduced if multiple individuals carry out chirp collection using the same signal, and if the GT set is assembled based on the overlap across sets collected by different individuals.

When chirps appear in the signal at a low frequency, the time needed for an individual to collect a sufficiently large GT set increases. While the validation of any algorithm requires the collection of all chirps from a test signal, the number of samples needed by our supervised-learning method is higher than the number of samples needed for validation only. Nevertheless, our method can still be advantageous compared to traditional approaches when already detected chirp types are expected in future experiments. In such cases, the cluster shapes from already collected GT sets can be reused. Furthermore, one can even build libraries of cluster shapes which can then be employed to “scan” signals for all formerly identified chirp shapes.

As input, our supervised learning method uses the time–frequency–amplitude signal $(T_k, f_k, A_k)$, $k=1,2, \ldots$. While the method for the computation of this signal, described in Sect. “Calculation of EOD frequency and amplitude”, works only for time–voltage data that were generated by a single EOD source, for the analysis of multiple (either synthetic or recorded from fish) simultaneously recorded EOD signals, one can employ a different method (e.g., Raab et al. 2022) to extract individual time–frequency–amplitude signals.

### Perspectives

The presented supervised learning algorithm provides a valuable tool for further examining the function of chirps. In the present study, it has not only enabled us to validate the previous classification of chirps into different subtypes, but also suggested that further differentiation of these subtypes is possible. Whether these sub-subtypes of chirps subserve any behavioral function remains to be examined.

It is likely that other algorithms based on supervised machine learning will exhibit advantages similar to our approach. Thus, the present study might serve as proof-of-principle of the suitability of the supervised-machine-learning paradigm for a broad range of signals analyzed in neuroethology. It is likely that, in future investigations, algorithms based on machine learning paradigms like the one implemented in the present study will increasingly become standard tools for signal analysis in neuroethological research.

## Data Availability

The code file implementing the supervised-learning algorithm is available in a public GitHub repository at the following link: https://github.com/LaboratoryOfNeurobiology/supervised_learning_of_chirp_patterns. Data that support the findings of this study are available from the corresponding author upon reasonable request.

## Appendix A: Computation of time-series frequency and amplitude data

Using linear interpolation, we first computed all time instances where the filtered time-series signal $\left( t_i, V_i\right)$, $i=1, \ldots , M_\textrm{v}$, crosses the time axis toward positive voltage values:

A1 $$\begin{aligned} \textbf{t}^+ = \left( t_{\textbf{j}(k)} - \frac{V_{\textbf{j}(k)}}{V_{\textbf{j}(k)+1}-V_{\textbf{j}(k)}}\left( t_{\textbf{j}(k)+1}-t_{\textbf{j}(k)}\right) : k=1, \ldots , M+1\right) . \end{aligned}$$

Here the tuple of all upward crossings $\textbf{t}^+$ contains $\left\vert \mathbf{j} \right\vert=M+1$ number of elements, with

A2 $$\begin{aligned} \textbf{j} = \left( i: \textrm{sgn}\left( V_{i+1}\right) -\textrm{sgn}\left( V_{i}\right) >0, i=1, \ldots , M_\textrm{v}\right) \end{aligned}$$

being a tuple containing all indices of the filtered time-series data where the voltage changed sign to a positive value. Finally, for each oscillation interval $\left[ \textbf{t}^+(k),\textbf{t}^+(k+1)\right]$, $k=1, \ldots , M$, we computed time instance $T_k$, and associated frequency $f_k$ and peak-to-peak amplitude $A_k$ values as

A3 $$\begin{aligned} T_k&= \frac{\textbf{t}^+(k+1)+\textbf{t}^+(k)}{2}, \end{aligned}$$

A4 $$\begin{aligned} f_k&= \frac{1}{\textbf{t}^+(k+1)-\textbf{t}^+(k)}, \end{aligned}$$

A5 $$\begin{aligned} A_k&= \underset{\textbf{j}(k)\le i \le \textbf{j}(k+1)}{\max }\!\left( v_i\right) - \underset{\textbf{j}(k)\le i \le \textbf{j}(k+1)}{\min }\!\left( v_i\right) . \end{aligned}$$
